# Hevin is down-regulated in many cancers and is a negative regulator of cell growth and proliferation

**DOI:** 10.1054/bjoc.1999.1051

**Published:** 2000-02-21

**Authors:** A Claeskens, N Ongenae, J M Neefs, P Cheyns, P Kaijen, M Cools, E Kutoh

**Affiliations:** Department of Biochemistry, Biotechnology, Immunology, Janssen Research Foundation, Turnhoutseweg 30, Beerse, B-2340, Belgium; Department of Surgery, St Jozef Hospital, Stw op Merksplas 44, Turnhout, B-2200, Belgium

**Keywords:** Hevin, cancer, tumor suppressor gene, cell cycle, cell proliferation

## Abstract

We have cloned a human Hevin cDNA from omental adipose tissue of different patients by reverse transcription polymerase chain reaction and shown a sequence variation due to a possible polymorphism at amino acid position 161 (E/G). Hevin protein expressed in vitro showed molecular weights of approximately 75 kDa and 150 kDa, suggesting that Hevin may form a homodimer in vitro. Using Northern blots and a human expressed sequence tAg database analysis, Hevin was shown to be widely expressed in human normal or non-neoplastic diseased tissues with various levels. In contrast to this, its expression was strongly down-regulated in most neoplastic cells or tissues tested. However, neither the mechanism nor the physiological meaning of this down-regulation is known. As an initial step towards investigating the functional role of Hevin in cell growth and differentiation, we transiently or stably expressed this gene in cancer cells (HeLa 3S) that are devoid of endogenous Hevin and measured DNA synthesis (cell proliferation) by 5-bromo-2′-deoxyuridine incorporation. Hevin was shown to be a negative regulator of cell proliferation. Furthermore, we have shown that Hevin can inhibit progression of cells from G1 to S phase or prolong G1 phase. This is the first report which describes the function of Hevin in cell growth and proliferation. Through database analysis, Hevin was found to be located on chromosome 4 which contains loss of heterozygosity of many tumour suppressor genes. Taken together, these results suggest that Hevin may be a candidate for a tumour suppressor gene and a potential target for cancer diagnosis/therapy. © 2000 Cancer Research Campaign


*British Journal of Cancer* (2000) **82**(6), 1123–1130
© 2000 Cancer Research Campaign
Article no. bjoc.1999.1051, available online at http://www.idealibrary.com on IDEAL®

### Hevin is down-regulated in many cancers and is a negative regulator of cell growth and proliferation

**A Claeskens[1], N Ongenae[1], JM Neefs[2], P Cheyns[4], P Kaijen[1], M Cools[3] and E Kutoh[1]**

Department of [1]Biochemistry, [2]Biotechnology and [3]Immunology, Janssen Research Foundation, Turnhoutseweg 30, B-2340 Beerse, Belgium; [4]Department of Surgery, St Jozef Hospital, Stw op Merksplas 44, B-2200 Turnhout, Belgium

**Summary** We have cloned a human Hevin cDNA from omental adipose tissue of different patients by reverse transcription polymerase chain reaction and shown a sequence variation due to a possible polymorphism at amino acid position 161 (E/G). Hevin protein expressed in vitro showed molecular weights of approximately 75 kDa and 150 kDa, suggesting that Hevin may form a homodimer in vitro. Using Northern blots and a human expressed sequence tAg database analysis, Hevin was shown to be widely expressed in human normal or non-neoplastic diseased tissues with various levels. In contrast to this, its expression was strongly down-regulated in most neoplastic cells or tissues tested. However, neither the mechanism nor the physiological meaning of this down-regulation is known. As an initial step towards investigating the functional role of Hevin in cell growth and differentiation, we transiently or stably expressed this gene in cancer cells (HeLa 3S) that are devoid of endogenous Hevin and measured DNA synthesis (cell proliferation) by 5-bromo-2′-deoxyuridine incorporation. Hevin was shown to be a negative regulator of cell proliferation. Furthermore, we have shown that Hevin can inhibit progression of cells from G1 to S phase or prolong G1 phase. This is the first report which describes the function of Hevin in cell growth and proliferation. Through database analysis, Hevin was found to be located on chromosome 4 which contains loss of heterozygosity of many tumour suppressor genes. Taken together, these results suggest that Hevin may be a candidate for a tumour suppressor gene and a potential target for cancer diagnosis/therapy. © 2000 Cancer Research Campaign

**Keywords**: Hevin; cancer; tumor suppressor gene; cell cycle; cell proliferation

Identification of cell regulatory genes that drive carcinogenesis (e.g. oncogenes, tumour suppressor genes and DNA repair genes) is one of the major goals in cancer research, eventually resulting in new anticancer drug targets and new therapeutic and diagnostic approaches. These genes are frequently found to be differentially expressed between normal and neoplastic tissues, suggesting that over-expression or loss of expression of these genes will lead to carcinogenesis (Weinberg, 1996). The Hevin gene was initially cloned from endothelial cells in lymphoid tissues and is thought to facilitate lymphocyte migration through the endothelium by modulating endothelial cell adhesion (Girard and Springer, 1996). Early evidence suggests that it could be an important regulatory gene in neoplastic tissues (Bendik et al, 1998; Nelson et al, 1998). Recently, Hevin expression was shown to be down-regulated in prostate (Nelson et al, 1998) and non-small-cell lung (Bendik et al, 1998) cancers, suggesting that Hevin may be involved in the pathogenesis of these cancers. Sequence analysis of the Hevin gene has revealed that it encodes a secreted, acidic, calcium-binding protein closely related to the anti-adhesive protein called SPARC (also known as osteonectin or BM-40; Girard and Springer, 1995). SPARC and Hevin share 62% identity over a region of 232 amino acid residues spanning more than four-fifths of the SPARC coding sequence. Although these two genes are related structurally and functionally, it is likely that the proteins have both distinct and overlapping physiological roles. For example, while Hevin is expressed in many tissues, SPARC expression is tissue-specific (Girard and Springer, 1996). Previous evidence indicates that SPARC may function as a negative regulator of cell proliferation by inhibiting the progression of cells from G1 to S phase (Funk and Sage, 1991). Support for this finding was provided in a study using differential display in which SPARC was identified as a down-regulated transcript in ovarian carcinoma (Mok et al, 1996). Genes that are negative regulators of cell proliferation (e.g. tumour suppressor genes) are frequently down-regulated in cancer tissues (Weinberg, 1995). However, Hevin expression in cancers other than prostate and lung or its physiological function in cell growth and differentiation remains completely unknown. In this paper, we report the molecular cloning of the human Hevin gene from omental adipose tissues and demonstrate mismatches with the published sequence. We have analysed its expression in many normal and neoplastic tissues and, furthermore, studied for the first time its effect in cell growth and proliferation.

*Received 9 February 1999*
*Revised 13 October 1999*
*Accepted 13 October 1999*

*Correspondence to:* E Kutoh

### MATERIALS AND METHODS

### Human tissues and preparation of RNA

Pieces of human omental adipose tissue (between 10 and 1000 g, five patients) were obtained during intra-abdominal surgery and immediately frozen in dry ice. The patients were men under the age of 65. Total RNA of the human tissues or cultured HeLa 3S cells was prepared using Trizol reagent (Life Technologies, Paisley, UK) as described previously (Kutoh et al, 1998).

### Cloning of human full-length Hevin cDNA by RT-PCR, sequencing and database analysis

One microgram of RNA was reverse-transcribed (RT) by 500 ng of the human Hevin reverse primer (sequence shown below; all the oligonucleotides used in this work were purchased from Eurogentec, Seraing, Belgium), 1 µl of 10 mM dNTPs and 200 units of SuperScrip II reverse transcriptase (Life Technologies) in a total volume of 20 µl according to the manufacturers' protocol. After the reaction, the mixture was heated at 65°C for 10 min and 80 µl water was added. Subsequently, polymerase chain reactions (PCR) reactions were performed in a total volume of 50 µl containing 3 µl of cDNA, 1 µl of 20 µM each specific human Hevin primers, 1 µl of 10 mM dNTPs and 2.5 U of Platinum *Taq* high fidelity DNA polymerase (Life Technologies).

Forward: AGTGGCTCTGAGTCCAGCCCCTTAC (corresponding to nt 156–180 from the human Hevin sequence; GenBank/EMBL accession no. X82157). Reverse: CGTTCAAAACAAGAGATTTTCATCTATGTC (nt 2170–2195 of X82157) After an initial denaturation at 93°C for 2 min, PCR was carried out in 30 cycles (step 1 to step 3) using Gene Amp 9700 (Perkin-Elmer, Norwalk, CT, USA) as follows:

step 1: 93°C for 1 min (denaturation)
step 2: 61°C for 1 min (annealing)
step 3: 72°C for 2 min (reaction)
step 4: 72°C for 7 min (final elongation).

The amplified product (2040 bps) was ligated into pGEM-T easy Vector (Promega, Madison, WI, USA) according to the manufacturer's protocol. The construct was sequenced on both strands by using vector primers T7 and Sp6 and insert specific primers every 300 bp. Sequencing reactions were run on an Applied Biosystems 377 XL DNA sequencer (Perkin-Elmer). Two clones termed G1 and G5 having three different amino acid (aa) sequences at positions 116, 161 and 419 (for sequence, see the results section) were further prepared by plasmid Maxi kit (Qiagen, Hilden, Germany). The G5 construct was digested with *Eco*RI and the insert containing the full-length Hevin was ligated into the *Eco*RI site of the pTRE expression vector (Clontech, Palo Alto, CA, USA), yielding the clone termed M8. It was further prepared using EndoFree Maxi kit (Qiagen) for the cell transfection study.

### Expression of Hevin in vitro and molecular weight determination

Human Hevin protein was expressed in vitro using the two Hevin clones (G1 and G5) in the TNT T7 quick coupled transcription/translation system (Promega) in the presence of $^{35}$S-methionine (Amersham, Aylesbury, UK) as described previously (Kutoh et al, 1992). Two microlitres of the translated protein in reticulocyte lysate were loaded onto an 8.5% sodium dodecyl sulphate polyacrylamide gel electrophoresis (SDS-PAGE) (Haeffner et al, 1995). After electrophoresis, the gel was dried and exposed to X-ray film (Amersham) overnight at room temperature.

### Database analysis

The LifeSeq® v. 4.5 human EST database (Incyte Pharmaceuticals Inc., Palo Alto, CA, USA) was analysed to provide an indication of the human Hevin gene expression in various tissues. For each cDNA library in LifeSeq®, per cent abundance of Hevin expressed sequence tags (ESTs) was calculated as the fraction of the number of ESTs matching the Hevin gene over the total number of ESTs. cDNA libraries containing Hevin ESTs were subsequently grouped per tissue and disease status (normal, neoplastic and non-neoplastic diseased). Per cent abundance was averaged to overcome cDNA library size differences, again per tissue and disease status. A total of 1367 Hevin ESTs from 361 cDNA libraries were collected for this analysis. Chromosome location of the Hevin gene was queried on the 'gene map' at the National Center for Biotechnology Information (Bethesda, MD, http://www.ncbi.nlm.nih.gov/genemap/).

### Northern blot analysis

Northern blot nylon membrane containing 2 µg of poly-adenosine (poly-A) RNA from various human normal and neoplastic tissues/cells was purchased from Clontech (Multiple Tissue Northern Blots) and Invitrogen (mRNA REAL Blots, Carlsbad, CA, USA). The PCR-amplified fragment of the Hevin gene was purified from agarose gel using agarase (Boehringer Mannheim, Mannheim, Germany). The purified fragment was radiolabelled with $^{32}$P-dATP using a random primer (Boehringer Mannheim). Then, 345 bp of the PCR-amplified fragment of glyceraldehyde phosphate dehydrogenase (GAPDH) (Kutoh et al, 1998) were used as a control probe. Hybridization of the membrane overnight at 65°C using Express-Hyb hybridization solution (Clontech) and washing of the membrane were performed according to the manufacturer's protocol (Clontech). The signals were visualized by autoradiography using Hyperfilm (Amersham).

### Cell culture, transfection and cell proliferation assays

HeLa 3S tet-off cells (Yin et al, 1996) were purchased from Clontech. The cells were cultured in Dulbecco's modified Eagle's medium (DMEM) (Life Technologies) supplemented with 10% fetal calf serum in a 75-cm$^2$ flask. Transient transfection of the Hevin clone in pTRE expression vector (designated M8, see above) using Lipofectin® (Life Technologies) was performed according to the manufacturer's protocol. With this method, 70–90% of the cells were shown to be transfected by using the β-gal staining method (Sambrook et al, 1989). The cells were further cultured in the presence of 1 µg ml$^{-1}$ doxycycline (Dox, Sigma, St Louis, MO, USA) for 24 h. Then 5000 cells were transferred (per well) to a 96-multiwell plate and they were further cultured in the absence of Dox for 16 h. Subsequently, the rate of cell proliferation was measured by 5-bromo-2′-deoxyuridine (BrdU) incorporation (4 h) using Cell Proliferation enzyme-linked immunosurbent assay (ELISA) BrdU kit (Boehringer Mannheim) as described previously (Law et al, 1996).

HeLa 3S tet-off cells stably transfected with the Hevin clone (M8) were created according to the manufacturer's protocol (Clontech). Equal numbers of cells (1000–5000) were transferred (per well) to a 96-multiwell plate and cultured in the absence of Dox for 48–96 h. The rate of cell proliferation was measured by the same Cell Proliferation ELISA BrdU kit as mentioned above (Boehringer Mannheim).

Hevin expression in the cultured HeLa 3S tet-off cells in the absence or presence of 1 µg ml$^{-1}$ Dox or in human omental adipose tissue (HWAT) was verified by RT-PCR using the Hevin-specific

© 2000 Cancer Research Campaign

**Table 1** Sequence variation of human Hevin amino acids

| aa position | X82157 | G1 | G5 |
|---|---|---|---|
| 116 | S | T | T |
| 161 | E | E | G |
| 419 | T | A | A |

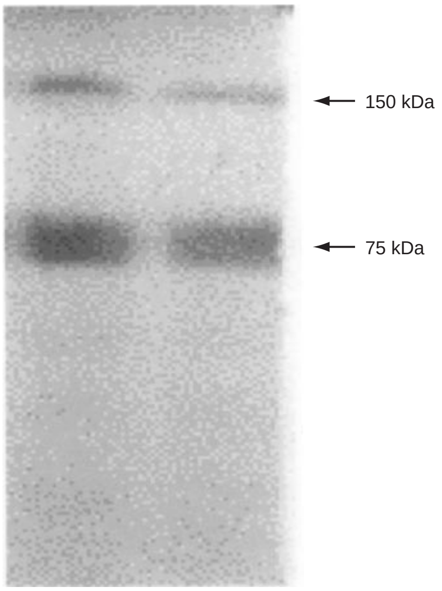

**Figure 1** Hevin may form a homodimer. Hevin was translated in vitro from two clones (G1 and G5) in the reticulocyte lysate in the presence of $^{35}$S-methionine as described in Materials and Methods. Two microlitres of the translated products were analysed by SDS-PAGE followed by autoradiography. The arrows indicate the molecular size of the translated products primers. Briefly, RNA was prepared at the same time as the BrdU ELISA assay. Then, 500 ng of the RNA was reverse-transcribed by oligo (dT) and the PCR reaction was performed as described above. The PCR products were loaded on 1.5% agarose 0.5 XTBE gel and the images of the ethidium bromide-stained gels were obtained using the Image Master (Pharmacia, Uppsala, Sweden). The PCR products were verified by sequencing.

### Cell cycle analysis

A total of $0.3 \times 10^6$ cells (stably transfected with Hevin or mock control) were seeded in 75-cm$^2$ flasks. The cells were cultured for 72 h in the absence of Dox. They were trypsinized (EDTA–trypsine, Life Technologies) and washed with phosphate-buffered saline (PBS). The number of cells was counted using Cytoron (Ortho, Raritan, NJ, USA). The cells were overnight fixed in ice-cold methanol and DNA staining was performed according to the method described by Vindelov et al (1983). PI-fluorescence of 10 000 nuclei was acquired on a FACS Calibur (Becton Dickinson, San Jose, CA, USA). Cell cycle analysis was done with Modfit software (Verity, ME, USA).

### RESULTS

### Full-length cDNA cloning of human Hevin and sequence analysis

Sequence analysis of the Hevin clone showed an open reading frame of 1992 nucleotides (nt). The predicted 664-aa Hevin protein has a calculated molecular weight of $M_r$ 75.2 kDa, with an isoelectric point of 4.68. There are three nt mismatches with the published human Hevin sequence (X82157) as assessed by the database analysis. These are at the nt positions 544 (T/A), 679 (A/G) and 1452 (A/G). Subsequently, they caused three aa changes at positions 116 (S/T), 161 (E/G) and 419 (T/A) as shown in Table 1. To study the consistency of these mismatches, we cloned the Hevin gene from five different patients. The mismatches at 116 aa and 419 aa were consistently found in tissues from five different patients. The mismatch at 161 aa, however, is controversial: three subjects had G, but the other two had the same sequence (E) as the published Hevin protein (Girard and Springer, 1996). We designated these two clones G1 (161 aa is E) and G5 (161 aa is G).

Hevin was further studied on the translational level. For this purpose, the two Hevin clones in pGEM-Teasy vector (designated G1 and G5) were translated in vitro in reticulocyte lysate in the presence of $^{35}$S-methionine, as described in Materials and Methods. Two major signals of 75 kDa and 150 kDa were obtained (Figure 1), suggesting that the Hevin protein may form a 150 kDa homodimer in vitro.

### Hevin is widely expressed in normal tissues but is strongly down-regulated in many cancers

Recent reports have indicated that Hevin expression is down-regulated in prostate cancer (Nelson et al, 1998) and non-small-cell lung cancer (Bendik et al, 1998). We have therefore addressed the question of whether this down-regulation is observed in other types of cancer. In order to answer this question, we first performed a Hevin expression analysis by a human EST database as described in Materials and Methods. A total of 1367 ESTs identical to the Hevin sequence were identified from 361 cDNA libraries from a wide range of tissues in the LifeSeq EST database (Incyte Pharmaceuticals). As shown in Figure 2, Hevin ESTs were found in a wide range of normal and non-neoplastic diseased tissues. Per cent abundance is especially high in artery, brain, fat, penis and uterus. However, in neoplastic tissues Hevin ESTs were found in only some tissues with lower per cent abundance (Figure 2, filled bar). These results suggest that the Hevin gene may be expressed at a higher level in non-neoplastic tissues (both normal and diseased) than in neoplastic tissues.

To confirm this database result experimentally, Northern blot analysis using a radiolabelled human Hevin probe and nylon membranes containing mRNAs from various human normal and cancer tissues/cells (Multiple Tissue Northern Blots, Clontech) was performed. As presented in Figure 3A panel I, a single signal of 2.8 kbps was constitutively observed with various levels in human normal tissues. High levels of expression were observed in heart, brain, skeletal muscle, small intestine and spinal cord. Low levels of expression were observed in liver and peripheral blood leucocyte. However, in contrast to this, Hevin expression in cancer cells was observed only in promyelocytic leukaemia (HL-60) and Burkitt's lymphoma (Raji) (Figure 3A, panel I). GAPDH was used as a control (Figure 3A, panel II).

*© 2000 Cancer Research Campaign*

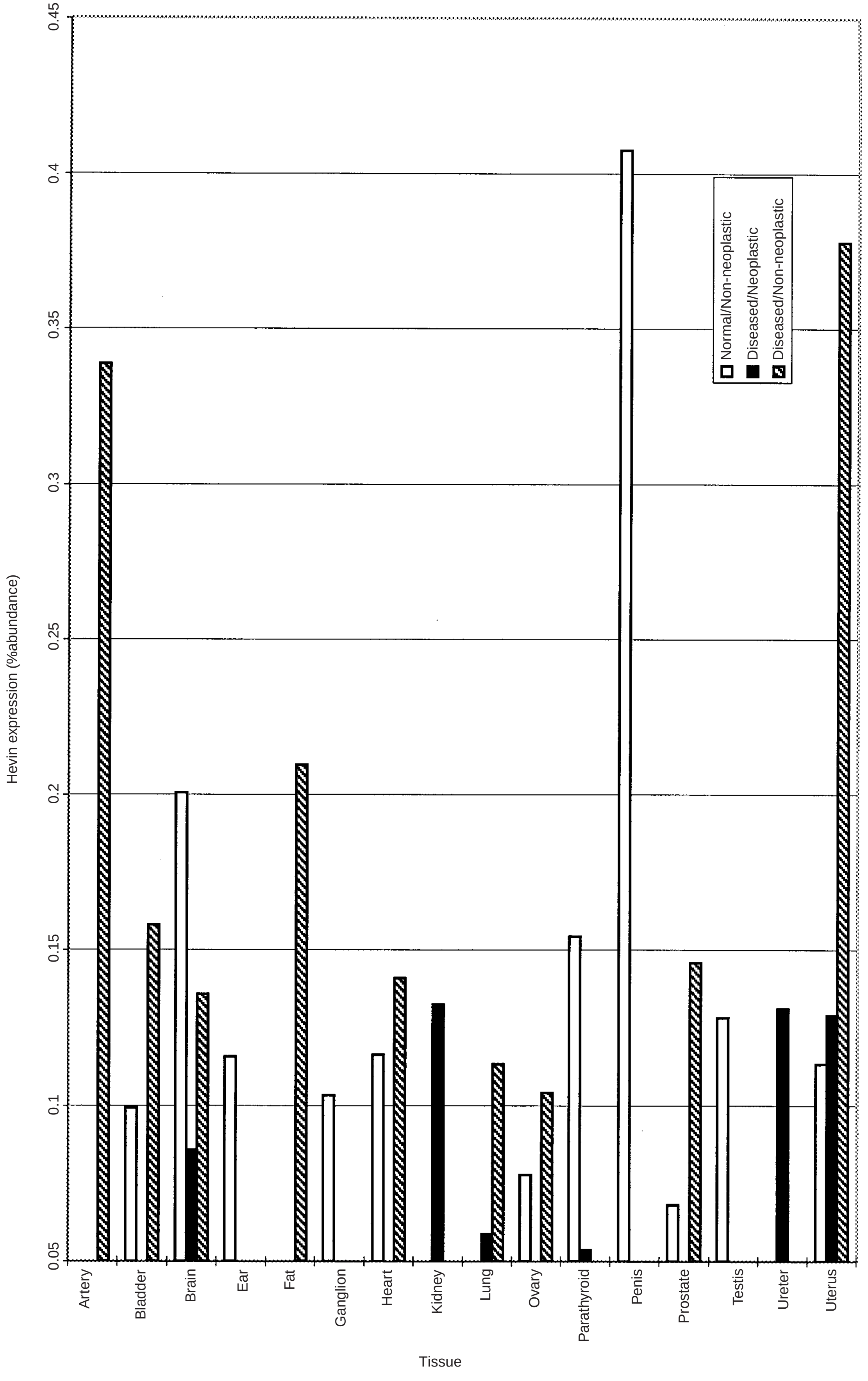

**Figure 2** Human EST analysis. The Hevin expression is estimated from per cent abundance (*y*-axis) of LifeSeq® database ESTs in cDNA libraries grouped according to tissue types and disease status (*x*-axis). Bars represent normal (open), neoplastic (filled) and non-neoplastic diseased (striped) tissues where the abundance of the Hevin ESTs is above 0.005%. Per cent abundance is defined as in Materials and Methods. No Hevin ESTs were found or the abundance was below 0.005% in tissues which show only one or two set of columns (e.g. artery or bladder)

*© 2000 Cancer Research Campaign*

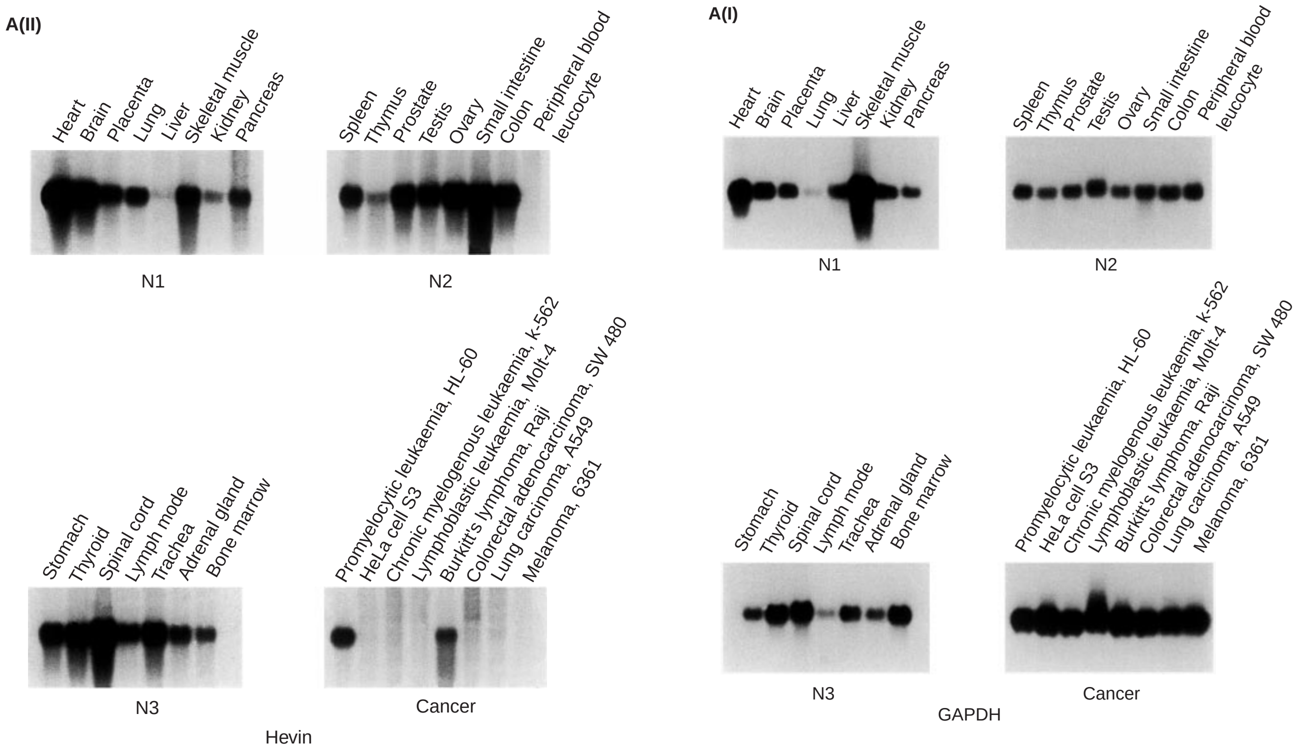

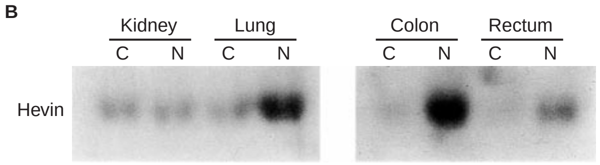

**Figure 3** Hevin expression is down-regulated in cancer tissues. (**A**) Northern membranes (MNT blots, Clontech) containing 2 μg of poly-A RNA from the indicated human normal tissues (N1–N3) and cancer cells (cancer) were hybridized with the radiolabelled human Hevin probe (panel I), then stripped and reprobed with a human GAPDH probe (panel II). (**B**) Northern membranes (mRNA real blots, Invitrogen) containing 2 μg of poly-A RNA from the indicated human normal (N) and cancer (C) patient-matched tissues were hybridized with the radiolabelled human Hevin probe

We extended this experiment using nylon membranes containing equal amounts (2 μg) of mRNA from various human tissues, both normal and cancerous (mRNA REAL Blots, Invitrogen). The quality of the membrane (RNA integrity, loading equality) was verified by the manufacturer. This membrane has the advantage that the Hevin expression can be directly compared between normal and cancerous tissues from identical patients. Hevin expression was observed with various levels in all the four normal tissues tested (Figure 3B, designated N). However, in cancerous tissues (Figure 3B, designated C), Hevin expression was down-regulated in colon, rectum and lung by comparison with those of normal counterparts. No difference was observed in kidney (Figure 3B). These results (Figure 3A, B) confirm that Hevin is widely expressed in normal tissues, but its expression is strongly down-regulated in many cancer cells/tissues.

### Hevin is a negative regulator of cell proliferation

The results obtained (Figure 3A,B) suggest that Hevin expression is strongly down-regulated in a wide range of neoplastic tissues. However, the physiological meaning of this phenomenon is unknown. Many genes that inhibit cell growth and differentiation (e.g. tumour suppressor genes) are also down-regulated in cancer cells (Bendik et al., 1998). In order to investigate the function of Hevin on cell growth and differentiation, we have transiently transfected the Hevin cDNA (M8, see Materials and Methods) using the tet-off System (Clontech) into HeLa 3S tet-off cells that lack the endogenous expression of Hevin (see Figure 3A). In this system, gene expression is turned on when doxycycline (Dox), an analogue of tetracycline, is removed (Gossen and Bujard, 1992; Gossen et al, 1995). Unlike other inducible expression systems, gene regulation in this system is very specific (Gossen et al, 1995). After the transfection, Hevin expression was verified using RT-PCR as described in Materials and Methods. Hevin transcript was clearly observed in the Hevin-transfected cells cultured in the absence of Dox. In contrast, this transcript was only weakly detected, if at all, in the cells that were transfected with the expression vector alone (pTRE) or in Hevin-transfected cells cultured in the presence of 1 μg $ml^{-1}$ Dox (results not shown). The rate of cell proliferation of the cells cultured in the absence of Dox was measured as described in Materials and Methods. As presented in Figure 4A, cells transfected with the Hevin clones showed a reduced level of cell proliferation (average 39.5%, 40.6% and 11.8% reduction in Experiments 1, 2 and 3, respectively) by comparison with those transfected with the mock vector (pTRE) alone.

In an effort to further prove the effect of Hevin on cell proliferation, we created HeLa 3S tet-off cells which were stably transfected

© 2000 Cancer Research Campaign 

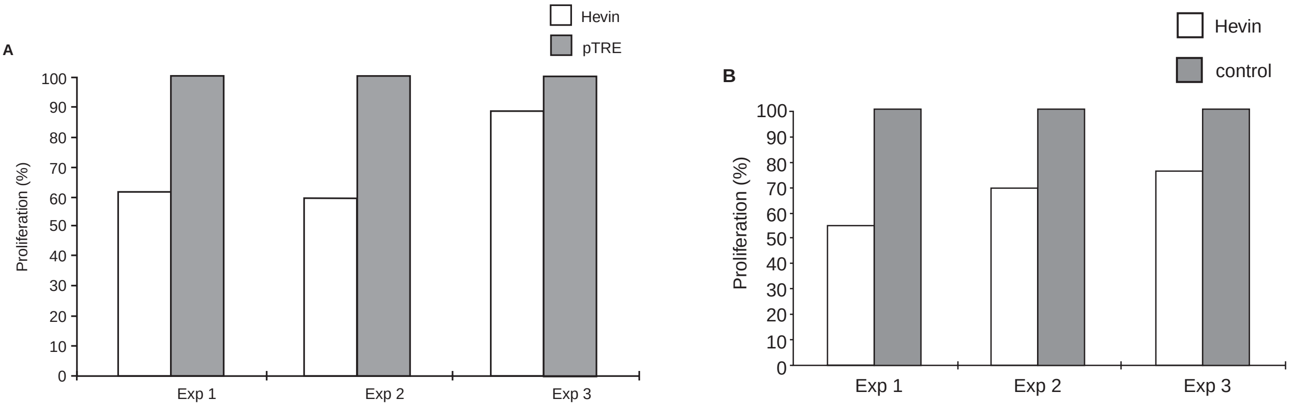

**Figure 4** Anti-proliferative effect of Hevin. (**A**) Transient expression. The rate of cell proliferation of transient transfected cells (Hevin and pTRE) was measured as described in Materials and Methods. The bar graphics represent the mean values obtained from the results of four different wells in each experiment. Values from the mock (pTRE) transfected cells were taken as 100%. Three independent experiments (Exp 1, Exp 2 and Exp 3) are presented. (**B**) Stable expression. The rate of cell proliferation of stable transfected or parental cells was measured as described in Materials and Methods. The bar graphics represent the mean values obtained from the results of four different wells in each experiment. Values from the parental cells were taken as 100%. Three independent experiments (Exp 1: 1000 cells per well cultured for 96 h, Exp 2: 2000 cells per well cultured for 96 h, Exp 3: 5000 cells per well cultured for 48 h) are presented with the Hevin clone. Cells were cultured in the absence or presence of 1 μg $ml^{-1}$ Dox. Hevin transcript was present in the transfected cells cultured in the absence of Dox. However, this transcript was only weakly observed in the transfected cells cultured in the presence of Dox or in the parental cells (results not shown). The rate of cell proliferation of the cells cultured in the absence of Dox was measured as described in Materials and Methods. As shown in Figure 4B, the transfected cells (indicated as Hevin) showed a reduced level (average 45.2%, 31.4% and 23.5% reduction in Experiments 1, 2 and 3 respectively) of proliferation by comparison with the control cells. These results suggest that, as in the transient expression data (Figure 4A), stably expressed Hevin can reduce the rate of cell proliferation.

In order to show that the observed effect of Hevin expression on cell proliferation (Figure 4) is specific and does not arise from very high (supra-physiological) levels or cytotoxic effects of Hevin, we measured: (i) levels of Hevin expression by comparison with those observed in human tissues and (ii) lactate dehydrogenase (LDH) from the culture medium as an indicator of cytotoxicity (Lagadic-Gossmann et al, 1998). Both in transiently and stably transfected cells, the level of exogenously expressed Hevin was not higher than those observed in human omental adipose tissues by using semi-quantitative RT-PCR (Figure 5). Furthermore, there was no difference in LDH concentrations in medium from the cells which do or do not express Hevin (results not shown). These results argue that the observed negative effect on cell proliferation (Figure 4, A, B) is caused specifically by the physiological function of Hevin.

### Hevin is involved in the cell cycle regulation

To study the effect of Hevin on cell cycle kinetics, we measured the proportion of cells in various stages of the cell cycle using FACScan flow cytometer. Equal numbers of cells that are stably transfected with Hevin or control cells were reseeded. After 3 days of culture, the number of the cells that express Hevin was approximately one-third of that of the cells that do not express Hevin (control), as presented in Table 2. When the distribution in each phase of the cell cycle was compared, the cells that express Hevin showed an increased proportion in G0/G1 phase but a reduction in S phase. In contrast to this, the percentage of G2/M phase was similar in both cells. This result suggests that Hevin inhibits the progression of cells from G1 to S phase or prolongs G1 phase.

### DISCUSSION

In this work, the human Hevin gene was cloned from human omental adipose tissue of different patients by RT-PCR and characterized. Sequence examination of our clones have revealed that they have three nucleotide mismatches with the published Hevin sequence (GenBank accession No: X82157). These mismatches cause amino acid changes in protein at positions 116 (S/T), 161 (E/G) and 419 (T/A) (Table 1). However, the changes at 116 aa (T) and 419 aa (A) were consistently found in five different patients and may therefore constitute the right sequence for Hevin. Alternatively, these sequence variations may be adipose tissue-specific. The variation at 161 aa (E/G) is controversial; out of five subjects, three had E and the others (two) had G. This mismatch

 © 2000 Cancer Research Campaign

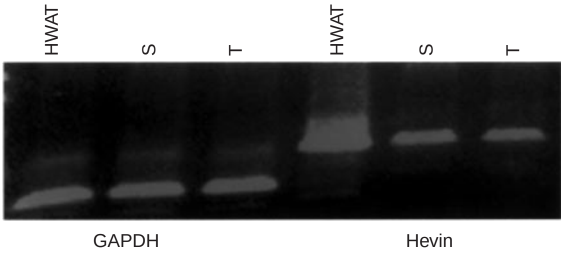

**Figure 5** Levels of Hevin expression in tet-off system and intact human tissue. RNAs were prepared from transiently (T) and stably (S) transfected HeLa 3S tet-off cells and human omental white adipose tissue (HWAT). Levels of Hevin expression were compared by quantitative RT-PCR using the Hevin-specific primer. GAPDH was used as a control

**Table 2** Effect of Hevin on HeLa cell cycle progression

| Cell phase | Distribution of cells (%) | | | Total cells[a] (% control) |
|---|---|---|---|---|
| | G0/G1 | S | G2/M | |
| Control | 52.3 | 30.5 | 16.9 | 100 |
| Hevin | 62.1 | 22.8 | 15.2 | 27 |

A total of $0.3 \times 10^6$ cells were grown for 72 h in 75-$cm^2$ flasks in the absence of Dox. Cell cycle measurements were performed as described in Materials and Methods. The given percentage corresponds to the mean of two samples. The experiment was repeated twice with similar results. [a]After 3 days, $2 \times 10^6$ cells are found in the control cultures.

may be due to a polymorphism of the Hevin gene at this position. However, we cannot rule out the possibility that these changes are caused by PCR mutations in spite of the fact that proof-reading Taq polymerase was used. However, these sequence variations do not lie in one of the potential functional domains of Hevin (transmembrane, acidic, follistin-like, or calcium binding domains) (Girard and Springer, 1995). Thus it is unlikely that these mismatches will affect the function of Hevin. To this end, sequence determination by screening different cDNA libraries could clarify the nature of these sequence variations. Alternatively, screening of meiosis (Lynch et al, 1995; Taylor et al, 1998) will determine whether these variations may be real polymorphisms.

We have further produced Hevin protein by in vitro translation in reticulocyte lysate and its protein appearance was determined by SDS-PAGE (Figure 1). The calculated $M_r$ of Hevin is 75 kDa. However, in addition to this signal, we have observed a 150 kDa signal. We therefore speculate that Hevin may form a homodimer in vitro. Indeed, Girard and Springer (1996) also observed that the $M_r$ 75 kDa Hevin migrated at a molecular weight of 130 kDa. Thus, homodimerization, a common theme for cell surface receptors, appears to explain the retained molecular mobility of Hevin. It remains to be tested whether Hevin forms a homodimer in vivo.

Based upon the Northern blots (Figure 3 A,B), Hevin was found to be expressed in a wide range of normal or non-neoplastic tissues, although with varying degrees. Conceivably, this high abundance in different tissues may indicate a more general non-tissue-specific role of Hevin. However, considering the tissue-specific degrees of expression (Figure 3 A,B), Hevin may play a more prominent role in tissues with high levels of expression (e.g. heart, skeletal muscle, small intestine, spinal cord; see Figure 3A). Initially, we cloned this gene from fat (omental adipose) tissues and we are currently interested in investigating the functional role of Hevin in adipogenesis and/or fat metabolism. Through the human EST database analysis (Incyte Pharmaceuticals, Palo Alto, CA, USA), Hevin ESTs were found at high frequency in inflammatory diseased tissues in artery and uterus, suggesting that Hevin may be involved in the inflammatory process (results not shown).

In contrast to this, Hevin expression is strongly down-regulated in cancer cell lines (Figure 3A) or in cancer tissues by comparison with matching normal tissues (Figure 3B). The mechanisms that cause this inactivation of its expression in neoplastic tissues have yet to be clarified. At least two hypotheses can be postulated for this down-regulation. One possibility is that the Hevin gene is deleted or mutated in cancer cells. Another possibility is that the gene is intact but its expression is impaired. Genetic and gene regulation studies are required in order to answer these questions. The fact that the Hevin expression is down-regulated in neoplastic tissues could be useful for diagnostic purposes. Furthermore, it is of interest to investigate at which stage of the cancer process this down-regulation occurs. This information will enhance diagnosis or determination of the staging of cancers.

Many genes responsible for tumorigenesis, for example, oncogenes and tumour suppressor genes, show a differential expression pattern between normal and neoplastic tissues. Some of these genes were cloned using this differential expression property (Nakshatri et al, 1996; Panotopoulou et al, 1997; Bendik et al, 1998). The tumour suppressor genes (or anti-oncogenes) are negative regulators of cell proliferation. They do so by regulating DNA synthesis, cell cycle checkpoints or apoptosis (Weinberg, 1995). Their expression and function are frequently lost in cancers (Weinberg, 1996). Due to the loss of expression of Hevin in cancer cells/tissues (Figure 3), we postulate that it may be a novel candidate tumour suppressor gene. Loss of function of tumour suppressor genes plays a central role in the development of cancer. The characterization of the biochemical pathways by which tumour suppressor alteration triggers tumorigenesis is the foundation for the design of novel therapeutic approaches (Fulci et al, 1998). Indeed, p53, the best studied tumour suppressor gene, has been successfully used in a gene therapy approach to cancer treatment and efforts are also being made to restore the p53 function using small molecules (reviewed in Chen and Mixson, 1998).

To investigate the functional role of Hevin in cell growth and differentiation, we expressed this gene transiently and stably using a tetracycline inducible expression vector (pTRE, Gossen et al, 1995) to the cells that lack endogenous expression of Hevin, and we measured the rate of proliferation (Figure 4 A,B). We were able to demonstrate that Hevin, in analogy to SPARC, is a negative regulator of cell proliferation. Further, we have shown that Hevin can inhibit the progression of cells from G1 to S phase (Table 2). To the best of our knowledge, this is the first report describing the effect of Hevin in cell growth and differentiation. This observation further supports our hypothesis that Hevin is a tumour suppressor gene. Tumour suppressor genes are known to inhibit cell growth and differentiation by regulating the entry of cells into the cell cycle, arresting the cell cycle progression, initiating programmed cell death (apoptosis). The detailed functional analysis of Hevin on those issues will be required for a better understanding of this gene. Loss of heterozygosity (LOH) analysis has been used in many types of human cancer to localize putative tumour suppressor genes. Through database analysis (National Center

© 2000 Cancer Research Campaign

Biotechnology Information, Gene Map), Hevin was mapped to chromosome 4. This chromosome has been shown to contain hot spots for LOH in many cancers (Obata et al, 1997; Radany et al, 1997; Ritland et al, 1997; Asamoto et al, 1998; Santos et al, 1998). This genetic approach again supports the notion that Hevin may be a tumour suppressor gene. Recently some tumour suppressor genes (e.g. APC, NF-2 and VHL) have been shown to be involved in cell–cell adhesion (Hirohashi, 1998; Ohh et al, 1998; Shaw et al, 1998). Proper cell–cell adhesion and communication are essential for normal cell growth and are often perturbed during cancer formation. Hevin, an anti-adhesive extracellular matrix protein, may exert its anti-proliferative effect through interference with cell–cell adhesion. In order to determine the physiological role of Hevin in vivo, and to further prove that it is indeed a tumour suppressor gene, it is particularly important to create a knock-out model of the Hevin gene. Ultimately, understanding Hevin at a molecular level and in vivo may uncover potential approaches for the treatment and diagnosis of cancers in humans.

### ACKNOWLEDGEMENTS

The authors thank Walter Wouters, Gerda Smets, Alan Richardson, Peter Roevens, Johan Auwerx and Didier De Chaffoy for discussions and critical reading of the manuscript and Peter Verhasselt for the sequencing work.

### REFERENCES

Asamoto M, Hori T, Baba-Toriyama H, Sano M, Takahashi S, Tsuda H and Shirai T (1998) p16 gene overexpression in mouse bladder carcinomas. *Cancer Lett* **127**: 9–13

Bendik I, Schraml P and Ludwig CU (1998) Characterization of MAST9/Hevin, a SPARC-like protein, that is down-regulated in non-small cell lung cancer. *Cancer Res* **58**: 626–629

Chen QR and Mixson JA (1998) Systemic gene therapy with p53 inhibits breast cancer: recent advances and therapeutic implications. *Front Biosci* **3**: 997–1004

Fulci G, Ishi N and Meir EG (1998) p53 and brain tumors: from gene mutations to gene therapy. *Brain Pathol* **4**: 599–613

Funk SE and Sage EH (1991) The $Ca^{2+}$ binding glycoprotein SPARC modulates cell cycle progression in bovine aortic endothelial cell. *Proc Natl Acad Sci USA* **88**: 2648–2652

Girard J-P and Springer TA (1995) Cloning from purified high endothelial venule cells of Hevin, a close relative of the antiadhesive extracellular matrix protein SPARC. Immunity **2**: 113–123

Girard J-P and Springer TA (1996) Modulation of endothelial cell adhesion by Hevin, an acidic protein associated with high endothelial venules. *J Biol Chem* **271**: 4511–4517

Gossen M and Bujard H (1992) Tight control of gene expression in mammalian cells by tetracycline-responsive promoters. *Proc Natl Acad Sci USA* **89**: 5547–5551

Gossen M, Freundlieb S, Bender G, Muller G, Hillen W and Bujard H (1995) Transcriptional activation by tetracyclines in mammalian cells. *Science* **268**: 1766–1769

Haeffner B, Baxter R, Fincham V, Downes P and Frame M (1995) Cooperation of Src homology domains in the regulated binding of phosphatidylinositol 3-kinase. *J Biol Chem* **270**: 7937–7943

Hirohashi S (1998) Inactivation of the E-cadherin-mediated cell adhesion system in human cancers. *Am J Pathol* 153: 333–339

Kutoh E, Stromstedt P-E and Poellinger L (1992) Functional interference between the ubiquitous and constitutive octamer transcription factor 1 (OTF-1) and the glucocorticoid receptor by direct protein–protein interaction involving the homeo subdomain of OTF-1. *Mol Cell Biol* **11**: 4960–4969

Kutoh, E, Boss, O, Levasseur, F and Giacobino, J-P (1998) Quantification of the full length leptin receptor (OB-Rb) in human brown and white adipose tissue. Life Sci **62**: 445–451

Lagadic-Gossmann D, Rissel M, Le Bot MA and Guillouzo A (1998) Toxic effects of tacrine on primary hepatocytes and liver epithelial cells in culture. *Cell Biol Toxicol* **5**: 361–373

Law RE, Meehan WP, Xi XP, Graf K, Wuthrich DA, Coats W, Faxon D and Hsueh WA (1996) Troglitazone inhibits vascular smooth muscle cell growth and intimal hyperplasia. *J Clin Invest* **98**: 1897–1905

Lynch TJ, Brickner J, Nakano KJ and Orias E (1995) Genetic map of randomly amplified DNA polymorphisms closely linked to the mating type locus of *Tetrahymena thermophila*. *Genetics* **141**: 1315–1325

Mok S, Chan WY, Wong KK, Muto MG and Berkowitz RS (1996) SPARC, an extracellular matrix protein with tumor-suppressing activity in human ovarian epithelial cells. *Oncogene* **12**: 1895–1901

Nakshatri H, Bouillet P, Bhat-Nakshatri P and Chambon P (1996) Isolation of retinoic acid-repressed genes from P19 embryonal carcinoma cells. *Gene* **174**: 79–84

Nelson PS, Plymate SR, Wang K, True LD, Ware JL, Gan L, Liu AY and Hood L (1998) Hevin, an anti-adhesive extracellular matrix protein is down-regulated in metastatic prostate adenocarcinoma. *Cancer Res* **58**: 232–236

Obata M, Lee GH, Kanda H, Kitagawa T and Ogawa K (1997) Loss of heterozygosity at loci on chromosome 4, a common genetic event during the spontaneous immortalization of mouse embryonic fibroblasts. *Mol Carcinog* **19**: 17–24

Ohh M, Yauch RL, Lonergan KM, Whaley JM, Stemmer-Rachamimov AO, Louis DN, Gavin BJ, Kley N, Kaelin WG, Jr and Iliopoulos O (1998) The von Hippel–Lindau tumor suppressor protein is required for proper assembly of an extracellular fibronectin matrix. *Mol Cell* **7**: 959–968

Panotopoulou E, Fidas A, Apostolikas N, Besbeas S, Papas T and Kottaridis D (1997) Isolation of a cDNA clone from colon carcinoma. *Anticancer Res* 3441–3444

Radany EH, Hong K, Kesharvarzi S, Lander ES and Bishop JM (1997) Mouse mammary tumor virus/v-Ha-*ras* transgene-induced mammary tumors exhibit strain-specific allelic loss on mouse chromosome 4. *Proc Natl Acad Sci USA* **94**: 8664–8669

Ritland SR, Rowse GJ, Chang Y and Gendler SJ (1997) Loss of heterozygosity analysis in primary mammary tumors and lung metastases of MMTV-MTAg and MMTV-neu transgenic mice. *Cancer Res* **57**: 3520–3525

Sambrook J, Fritsch E and Maniatis T (1989) *Molecular Cloning: A Laboratory Manual*, 2nd edn. Cold Spring Harbor Laboratory Press, NY, USA

Santos J, Herranz M, Perez de Castro I, Pellicer A and Fernandez-Piqueras J (1998) A new candidate site for a tumor suppressor gene involved in mouse thymic lymphomagenesis is located on the distal part of chromosome 4. *Oncogene* **17**: 925–929

Shaw RJ, McClatchey AI and Jacks T (1998) Regulation of the neurofibromatosis type 2 tumor suppressor protein, merlin, by adhesion and growth arrest stimuli. *J Biol Chem* **273**: 7757–7764

Taylor JF, Coutinho LL, Herring KL, Gallagher DS Jr, Brenneman RA, Burney N, Sanders JO, Turner JW, Smith SB, Miller RK, Savell JW and Davis SK (1998) Candidate gene analysis of GH1 for effects on growth and carcass composition of cattle. *Anim Genet* **29**: 194–201

Vindelov LL, Christensen IJ, Keiding N, Spang-Thomsen M and Nissen NI (1983) Long-term storage of samples for flow cytometric DNA analysis. *Cytometry* **3**: 317–322

Weinberg RA (1995) The molecular basis of oncogenes and tumor suppressor genes. *Ann NY Acad Sci* 1995 **758**: 331–338

Weinberg RA (1996) How cancer arises. *Sci Am* 275: 62–70

Yin DX, Zhu L and Schimke RT (1996) Tetracycline-controlled gene expression system achieves high-level and quantitative control of gene expression. *Anal Biochem* **235**: 195–201

 © 2000 Cancer Research Campaign
